# Comparative Transcriptomic Analysis Reveals Sugar Metabolism and Accumulation Mechanisms in *Akebia trifoliata* Fruits

**DOI:** 10.3390/life16091459

**Published:** 2026-08-31

**Authors:** Juan Niu, Haili Zhu, Zhimin Sun, Yuanhe Wei, Guifa Cheng, Mingbao Luan

**Affiliations:** 1 School of Biological and Environmental Engineering, Jingdezhen University, Jingdezhen 333032, China; niujuan@jdeu.edu.cn (J.N.); weiyuanhe@jdzu.edu.cn (Y.W.); 2 National Nanfan Research Institute (Sanya), Chinese Academy of Agricultural Sciences, Sanya 572000, China; 3 School of Tropical Agriculture and Forestry, Hainan University, Danzhou 571737, China; 13917517960@163.com; 4 Institute of Bast Fiber Crops, Chinese Academy of Agricultural Sciences, Changsha 410205, China; sunzhimin@caas.cn; 5 Fuliang County Agricultural and Rural Bureau, Jingdezhen 333403, China; flxnyncj@163.com

**Keywords:** *Akebia trifoliata* pulp, fruit developmental stages, RNA-seq, sugar content

## Abstract

Sugar composition is a critical determinant of fruit quality in fleshy fruits, influencing both organoleptic properties and nutritional value. *Akebia trifoliata*, an emerging fruit crop, possesses pulp rich in soluble sugars, vitamins, and polyphenols, making it suitable for fresh consumption, medicinal applications, and food processing. However, the molecular mechanisms governing sugar metabolism and accumulation in this species remain largely unexplored. We systematically evaluated the sugar content dynamics across different *A. trifoliata* germplasms and performed comparative transcriptomic analysis between high-sugar-accumulation (S4) and low-sugar-accumulation germplasms (S19) at three developmental stages (IM: the fruit is hard and immature; MA: the pulp remains firm, but the peel begins to soften and crack; and PM: the fruit is fully mature, and the pulp becomes shiny, translucent, soft, and edible). A total of 10,269 differentially expressed genes were identified with KEGG enrichment analysis, revealing significant enrichment in pathways directly associated with sugar biosynthesis. Through the analysis of weighted gene co-expression networks, a co-expression module significantly correlated with total sugar accumulation was identified, and qRT-PCR was used to verify that the expression levels of 29 candidate genes involved in carbohydrate synthesis were higher in the high-sugar-accumulation type than in the low-sugar-accumulation germplasm. These findings not only advance our understanding of the molecular basis of fruit quality development in this underutilized crop but also lay the foundation for the future breeding of *A. trifoliata* varieties with high-sugar greater nutritional value and processing qualities for the food industry.

## 1. Introduction

Fruit quality is fundamentally shaped by endogenous metabolic processes, with soluble sugars serving as primary determinants of sweetness, flavor complexity, and consumer preference [1,2]. In the context of food science and the development of functional food ingredients, understanding the molecular basis of sugar accumulation in fruit crops has gained increasing importance. The composition and content of soluble sugars—particularly glucose, fructose, and sucrose—directly influence both organoleptic properties and nutritional value, thereby determining the suitability of fruits for fresh consumption, processing, and value-added product development.

Recent advances in high-throughput sequencing and multi-omics technologies have enabled the comprehensive dissection of sugar metabolism and its regulatory networks during fruit development. Integrative approaches combining transcriptomics with metabolomics or biochemical analyses have proven particularly powerful [3]. For instance, in *Rosa roxburghii*, combined transcriptome-metabolome profiling revealed dynamic patterns of major sugars (sucrose, glucose, and fructose) and identified multiple structural genes and transcription factors involved in sugar biosynthesis and transport [4]. Similarly, transcriptomic analysis in *Castanea mollissima* during embryo development enabled the construction of a sugar accumulation network, demonstrating sucrose predominance at the mature stage and implicating several regulatory factors [5]. In watermelon, comparative transcriptomic analyses of cultivars with contrasting sugar levels have identified differentially expressed genes (DEGs) associated with the accumulation of sugars and organic acids [6]. In grape, evidence synthesized from transcriptomics, metabolomics, and isotope-tracing studies has been used to systematically establish a framework linking carbon allocation, sugar accumulation, and acidity dynamics, and to pinpoint a series of regulatory nodes [7]. In *Vaccinium* fruits, integrated transcriptome–metabolome analyses have revealed the coordinated metabolic reprogramming of sugar, acid, and anthocyanin pathways during fruit development; sustained carbon import supports sugar accumulation and shapes fruit quality [8]. Collectively, these studies underscore that sugar accumulation in fruit flesh is governed not by single genes but by complex, coordinated networks [4,5,9]. Moreover, the stage-dependent expression patterns of sugar metabolism-related genes generally correlate closely with the sugar content dynamics [10]. Consequently, for underexplored plant species, especially *Akebia trifoliata*, the transcriptome-based investigation of sugar and sugar metabolism across developmental stages can provide species-specific insights into metabolic regulation while establishing a foundation for quality improvement, cultivation optimization, and domestication breeding [11].

*A. trifoliata* is a perennial woody vine belonging to the family Lardizabalaceae, characterized by berry-like fruits that transition from green to purplish-brown during ripening [12,13,14]. The fruit contains white, translucent pulp with a high nutritional value, characterized by a sweet taste and rich content of diverse sugars and bioactive compounds [15]. In recent years, this species has attracted growing attention in the food, medicinal, and functional product sectors [16]. During fruit ripening, the total soluble solids and total sugar contents increase progressively, with reported average total sugar levels of approximately 14.68 g/100 g fresh weight [17]. Despite its promising nutritional profile and application potential, the molecular mechanisms governing sugar metabolism and accumulation in *A. trifoliata* fruits remain largely unexplored.

At present, reports on specific enzymes and transporters involved in sugar metabolism in *A. trifoliata* are still limited. Only preliminary transcriptomic studies in *A. trifoliata* have indicated that late-stage ripening is accompanied by the marked up-regulation of sucrose-hydrolyzing enzymes, promoting sucrose cleavage into fructose and glucose and thereby enhancing pulp sweetness [18]. This study provides a basis for the coordinated expression of sugar transport pathways during starch/polysaccharide degradation, sucrose synthesis and hydrolysis, and maturation using transcriptomics methods. Therefore, systematic transcriptome analysis can not only reveal the expression dynamics of key genes in the sugar metabolism pathway—across starch degradation, sucrose hydrolysis, and fructose accumulation—but also construct an integrated network containing sugar metabolism, transport, and regulatory signals [19,20].

The fruit of *A. trifoliata* has important economic and medicinal value. Understanding the physiological and molecular mechanisms of its development and maturation is crucial for improving fruit quality, extending shelf life, and advancing breeding programs. Identifying key genes and co-expression modules related to sugar accumulation provides targets for transforming wild fruit resources into industrial-related applications, which, in turn, can improve the sweetness and nutritional quality of fruits through molecular breeding or genome editing, thereby improving market competitiveness and supporting the development of functional foods and value-added products [12,20]. As the main determinant of fruit flavor, little is known about the molecular mechanism of sugar metabolism regulation in *A. trifoliata* fruit. In this study, based on the comparative analysis of the sugar content in fruits of different germplasms of *A. trifoliata* at the mature stage, we selected two germplasms of low sugar and high sugar for transcriptome (RNA-seq) analysis. The weighted gene co-expression network analysis (WGCNA) identified co-expression modules related to sugar accumulation, from which 29 differentially expressed genes related to sugar metabolism were identified. In conclusion, this study comprehensively elucidated the regulation mechanism of sugar metabolism in *A. trifoliata*, which laid a foundation for formulating targeted genetic improvement strategies and strengthening the development and utilization of this valuable germplasm resource. In addition, these results can be used as a reference model for studying the sugar accumulation mechanism of other underutilized berry crops, and promote the wider use of local fruit resources in nutrition, health, and industrial applications.

## 2. Materials and Methods

### 2.1. Sample Collection

In this study, 26 8-year-old *Akebia trifoliata* germplasm resources in the nursery of the Institute of Bast Fibers, Chinese Academy of Agricultural Sciences (Yuanjiang City, Hunan Province, 112°22′ E, 28°49′ N) were used as research materials. As a reference, Red Fuji apple, Hainan banana, and Kyoho grape are commercial mature fruits purchased from supermarkets. Each variety buys nine fruits, and three are biological replicates. Among them, 3 grapes were taken from 9 clusters, and 3 clusters of fruits were biological replicates. The samples were biological replicated three times for subsequent analysis. When the fruit of *A. trifoliata* is immature (IM, 135 days after flowering), the fruit is hard and the peel is closed. As the fruit begins to ripen, the pulp remains firm, but the peel begins to soften and cracks longitudinally along the ventral suture (MA, 145 days after flowering). When the fruit is fully mature, the flesh becomes shiny, translucent, soft, and edible, accompanied by complete cracking of the peel (PM, 155 days after flowering) of the peel. Three fruits were randomly collected from each tree every 10 days during the fruit development period (12 September, 22 September, 2 October 2024) and mixed into a biological replicate. The fruits of 9 trees were collected as 3 biological replicates for further analysis. The pulp was rapidly dissected in the fruit, immediately frozen in liquid nitrogen, and stored at −80 °C until used for sugar content, transcriptome, and qRT-PCR analysis.

### 2.2. Determination of Sugar Content in the Pulp of A. trifoliata Fruit

For each of the 26 *A. trifoliata* germplasms and the common fruits (apple, banana, and grape), the following procedure was performed. The determination of fructose, sucrose, and glucose in the pulp of samples were referenced to the previously published method with slight modifications [21,22,23]. The frozen sample to be tested was ground and added with 10 mL 80% ethanol to fully grind to form a homogeneous slurry. The milled slurry was transferred to a centrifuge tube, incubated in a water bath at 80 °C for 40 min, and then 2 g activated carbon was added for decolorization for 20 min. After centrifugation, the supernatant was transferred to the volumetric flask. The residue was subjected to the same procedure and the supernatant was combined. Finally, the volume was fixed with distilled water for later use. The contents of fructose, sucrose, and glucose in the samples were determined, respectively, and the sum of the three was taken as the total sugar content for analysis.

Three independent colorimetric assays were performed to quantify fructose, glucose, and sucrose in the diluted extracts (prepared as described above). All measurements were conducted in triplicate, and the mean values were used for standard curve calculations.

For fructose determination, an aliquot of 0.1 mL of the diluted extract was mixed with 3 mL of anthrone reagent in a glass test tube, with a blank control prepared in parallel. The mixture was incubated in the dark at room temperature, and the absorbance was subsequently read at 620 nm. A standard curve was constructed using anhydrous fructose (10 mg dissolved in 100 mL sterile water), from which 0, 0.1, 0.2, 0.4, 0.6, 0.8, and 1.0 mL aliquots were taken, adjusted to 2 mL with sterile water, and processed identically.

For glucose (reducing sugar) quantification, a 0.5 mL aliquot of the diluted extract was reacted with 1 mL of 3,5-dinitrosalicylic acid (DNS) reagent. The mixture was heated in a boiling water bath for 2 min, rapidly cooled in running cold water, and then diluted with 4.5 mL of distilled water. Absorbance was measured at 540 nm. A standard curve was generated using anhydrous glucose (100 mg in 100 mL sterile water), with aliquots (0–1.0 mL) adjusted to 2 mL with sterile water and processed under the same conditions, with the final volume adjusted to 5 mL prior to absorbance reading.

For sucrose measurement, a 0.1 mL aliquot of the diluted extract was first incubated with 0.1 mL of 30% KOH in a boiling water bath for 10 min. After cooling to room temperature, 3 mL of anthrone reagent was added, and the mixture was incubated at 40 °C for 15 min, followed by absorbance measurement at 620 nm. The sucrose standard curve was prepared from anhydrous sucrose (10 mg in 100 mL sterile water), using the same aliquot series and processing as described for fructose, except for the initial KOH hydrolysis step.

All standard curves were plotted as absorbance versus corresponding sugar concentration, and sample concentrations were calculated by interpolation from the respective standard curves. The concentrations of fructose, glucose, and sucrose in the samples were determined by interpolating their respective absorbance values (As) into the corresponding standard curves. For each sugar, the content was calculated using the following equation:

Sugar content (mg/g FW) = (As − b) × Vt × D/(k × W)

where:

As = absorbance of the sample;
b = intercept of the standard curve;
k = slope of the standard curve;
Vt = total volume of the extract (mL);
D = dilution factor (specific to each assay protocol);
W = fresh weight of the sample (g).

The total soluble sugar content was then obtained by summing the individually calculated amounts of fructose, glucose, and sucrose.

### 2.3. RNA Extraction, cDNA Library Construction, and Illumina HiSeq Sequencing

Total RNA was extracted from fruit flesh of two *A. trifoliata* genotypes (S4 and S19) at three developmental stages (IM, MA, and PM), with three biological replicates per sample (18 libraries in total), using the mirVana™ miRNA Isolation Kit (Cat. AM1561; Invitrogen, Thermo Fisher Scientific Inc., Carlsbad, CA, USA). Genomic DNA was removed by DNase I (Takara) treatment following the manufacturer’s instructions. RNA integrity was evaluated using an Agilent 2100 Bioanalyzer, and only samples with an RNA Integrity Number (RIN) ≥ 7 were used for library construction; the RIN values for all 18 libraries are provided in Table S1. RNA purity and concentration were assessed using a NanoDrop 2000 spectrophotometer (Thermo Fisher Scientific, Waltham, MA, USA) [24]. Sequencing libraries were constructed using the TruSeq Stranded mRNA LT Sample Prep Kit (Illumina, San Diego, CA, USA), with poly(A) mRNA enriched via oligo (dT) magnetic beads. Strand-specific libraries (dUTP method, RF orientation) were prepared and sequenced on an Illumina HiSeq X Ten platform with a 150 bp paired-end (PE150) configuration by OE Biotech Co., Ltd. (Shanghai, China). Raw paired-end reads in FASTQ format were processed using Trimmomatic v0.36 (http://www.usadellab.org/cms/?page=trimmomatic, accessed on 31 August 2026) [25] with parameters LEADING:3 TRAILING:3 ILLUMINACLIP:TruSeq3-PE-2.fa:2:30:10:8:true SLIDINGWINDOW:4:15 MINLEN:50 to remove adapters and low-quality bases. Quality assessments before and after trimming were performed using FastQC v0.11.5 and RSeQC v2.6.4, respectively.

The clean reads were then aligned to the *A. trifoliata* reference genome (Accession: PRJNA750300; BioSample: SAMN20447974; Sample name: M067-genomic; SRA accession: SRR15276322) using HISAT2 v2.2.1.0 [26] with parameters --rna-strandness rf --fr. For gene-level expression quantification, StringTie v1.3.3b (--rf) was used to compute FPKM values, and HTSeq-count v0.9.1 (-s reverse, default union mode) was used to obtain raw read counts per gene; reads that overlapped multiple genes ambiguously were excluded from counting. Differential expression analysis was performed on the raw count matrix using the DESeq R package v1.18.0, with genes considered significantly differentially expressed at a threshold of adjusted *p* < 0.05 and |log_2_Fold change| > 1. Alignment and count statistics are summarized in Table S2. The transcriptome data have been deposited in the National Center for Bio-technology Information (NCBI) database (https://dataview.ncbi.nlm.nih.gov/object/PRJNA1514214?reviewer=66grlb79q5orf4aiqo3df2h7sr, accessed on 19 August 2026), (BioProject: PRJNA1514214).

### 2.4. Differential Gene Expression Analysis

To identify differentially expressed genes among samples, FPKM (fragments per kilobase of transcript per million mapped reads) values were computed using StringTie v1.3.3b solely for expression visualization. Differential expression analysis was performed on the raw integer count matrix generated by HTSeq-count v0.9.1 using the DESeq R package v1.18.0 [27], which employs a negative binomial model with empirical-Bayes shrinkage for dispersion and fold-change estimation. The estimateSizeFactors function was used for data normalization, and the nbinomTest function was applied to compute *p*-values and fold changes for each pairwise comparison (S4_IM vs. S19_IM, S4_MA vs. S19_MA, and S4_PM vs. S19_PM). Independent filtering was applied to remove low-count genes prior to multiple testing, and the Benjamini–Hochberg procedure was used to control the false discovery rate. Genes with|log2 foldchange| > 1 and an adjusted *p*-value < 0.05 were considered significantly differentially expressed. All identified differential expressed genes (DEGs) were mapped to the Gene Ontology (GO) database (http://www.geneontology.org/, accessed on 31 August 2026) and KEGG database (http://www.kegg.jp/kegg/, accessed on 31 August 2026) to determine the main biological functions of DEGs and to identify significantly enriched metabolic and signal transduction pathways.

### 2.5. Gene Co-Expression Network

Weighted gene co-expression network analysis (WGCNA) was performed using the WGCNA R package v1.73 [28,29] in R 4.4.3. The input expression matrix consisted of log_2_(x + 1)-transformed FPKM values from all 18 samples, using genes with FPKM > 1 in at least 50% of samples. Among these, the top 50% of genes with the highest median absolute deviation (MAD) were retained for network construction, yielding 9104 genes for subsequent analysis. To determine the soft-thresholding power, the pickSoftThreshold function was applied across a range of powers (1–30) using Pearson correlations. The scale-free topology fit index (R^2^) and mean connectivity were calculated for each power; the lowest power achieving R^2^ > 0.77 was selected (β = 24, as shown in Figure S1). An unsigned network was constructed, and the adjacency matrix was transformed into a topological overlap measure (TOM) matrix. Hierarchical clustering was performed using the dynamic tree cut method (deepSplit = 2, minModuleSize = 30), and modules with similar expression profiles were merged at a cut height of 0.25. Grey represents unassigned genes that are not assigned to any module, and these unassigned genes are excluded from the downstream module-trait association analysis. Module eigengenes (first principal component of each module) were calculated and correlated with four sugar traits (fructose, glucose, sucrose, and total sugar) using Pearson correlation, with Student-approximated *p*-values. For each module, module membership (kME) was defined as the correlation between each gene’s expression profile and the module eigengene, and intramodular connectivity (kWithin) was calculated as the sum of adjacency strengths of a given gene to all other genes within the same module. Hub genes within each module were ranked by kWithin. A full gene-to-module assignment table for all 9104 input genes is provided as Supplementary Table S3.

### 2.6. Quantitative RT-PCR (qRT-PCR) Analysis

Total RNA was extracted from the fruit flesh of S4 and S19 using the SteadyPure Plant RNA Extraction Kit (Aikery, Changsha, China) according to the manufacturer’s instructions. First-strand cDNA was synthesized using the Evo M-MLV RT Premix for qPCR kit (Aikery). Primers were designed with Primer 5 software and synthesized by Tsingke Biotechnology (Beijing, China); all primer sequences are listed in Table 1. qRT-PCR was performed using 2× SYBR Green qPCR Mix (Aidlab, Beijing, China), and the reaction system and cycling program are provided in Table 2. In this study, the *EF-1α* gene was used as the internal control gene, which was detected by the de novo transcriptome sequencing of *A. trifoliata* [14]. Amplification was conducted on a Bio-Rad CFX96 Touch Real-Time PCR Detection System (Bio-Rad, Hercules, CA, USA) with three biological replicates [24]. A melting-curve analysis was generated at the end of each run for each sample. Raw data were processed in Excel 2016, and relative gene expression levels were calculated using the 2^−ΔΔCT^ method.

### 2.7. Statistical Analysis

GraphPad Prism 8 was used for data analysis and presentation. Three biological replicates were collected for each sample and the data in the figures were plotted with mean ± standard deviation. One-way ANOVA SPSS 21.0 software (Chicago, IL, USA) was used for statistical analysis. Duncan’s multiple-range test was used to determine the significant difference when *p* < 0.05. The asterisk represents the extremely significant difference between different samples (**: *p* < 0.01; ***: *p* < 0.001). Different lowercase letters (a–g) indicate significant differences at *p* < 0.05, arranged in ascending order of mean values (a < b < c < … < g).

## 3. Results

### 3.1. Analysis of Changes in Sugar Contents

**Figure 1:**
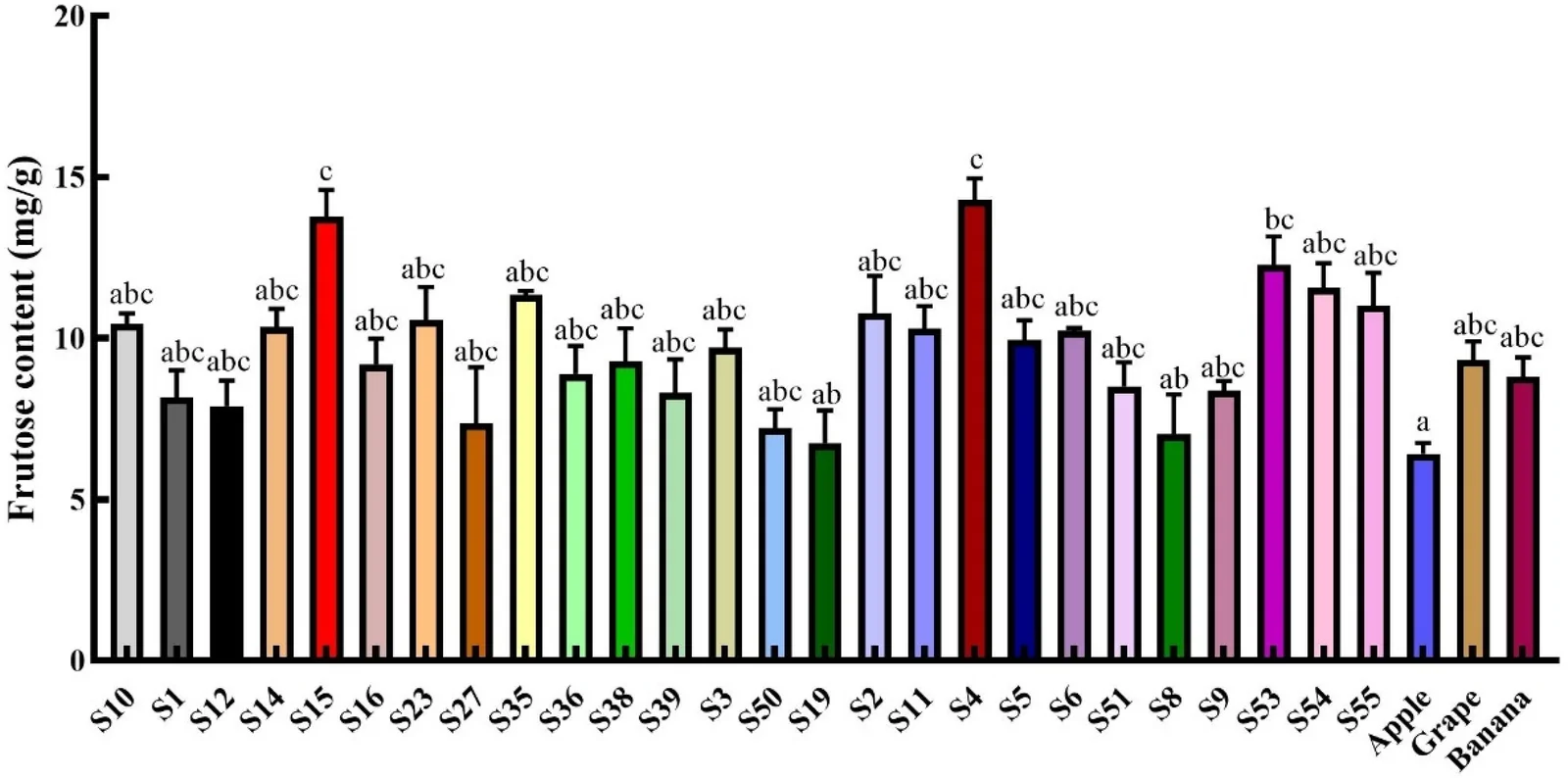
Contents of fructose of 26 different germplasms. Different lowercase letters (a–c) indicate significant differences at *p* < 0.05, arranged in ascending order of mean values (a < b < c).

The major sugars identified in the pulp of *A. trifoliata* were fructose, glucose, and sucrose. Research indicates that soluble sugars consist primarily of fructose, and reducing sugars account for 90.19% of the total sugar content, giving fresh *A. trifoliata* fruit a distinctly sweet taste [30,31]. Firstly, the fructose content in 26 *A. trifoliata* germplasms and 3 common fruits (banana, apple, and grape) was determined. Compared with the three common fruits, the fructose content in the 26 germplasms was higher than that in apples, and there was no significant difference in the fructose content between bananas and grapes. Notably, S15 and S4 germplasms showed the highest fructose contents, whereas cultivars S19 and S8 exhibited the lowest fructose contents among the tested *A. trifoliata* germplasms (Figure 1). To examine the accumulation patterns of major sugars across developmental stages, we measured the sugar contents in two high-sugar *A. trifoliata* cultivars (S4 and S15) and two low-sugar cultivars (S8 and S19). The results of the variance analysis are shown in Table S4.

**Figure 2:**
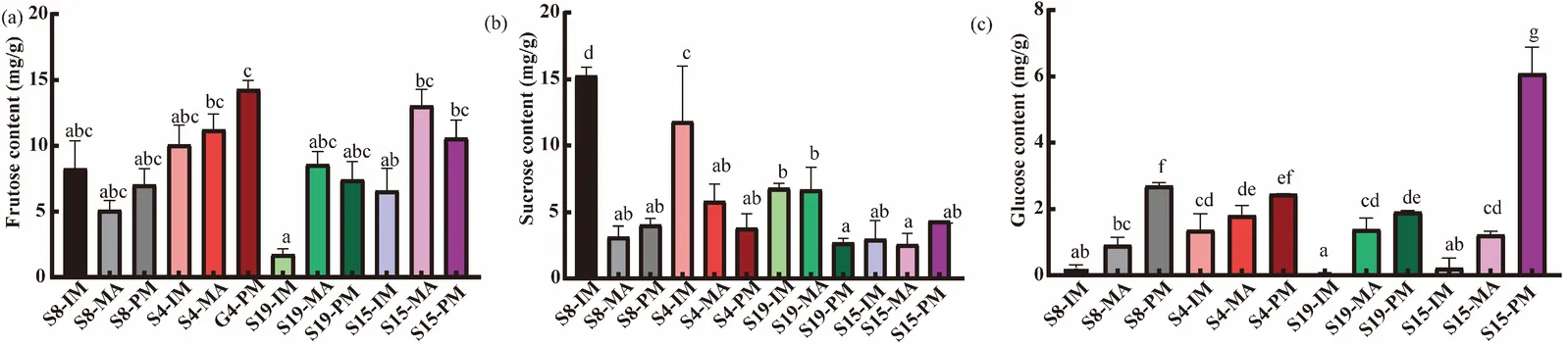
Contents of fructose (**a**), sucrose (**b**), and glucose (**c**) in the fruit flesh of four *A. trifoliata* cultivars at three developmental stages. Different lowercase letters (a–g) indicate significant differences at *p* < 0.05, arranged in ascending order of mean values (a < b < c < … < g).

Significant differences in the major sugar contents were observed among cultivars and across developmental stages. Cultivar S4 consistently exhibited the highest fructose content at all three developmental stages; notably, at the complete cracking stage (PM), its fructose content was significantly higher than that of the other germplasms. In the S4 germplasm, the fructose content was the lowest in the immature stage (IM), showing a pronounced difference compared with the later stages (Figure 2a). Germplasm S8 displayed a relatively high sucrose content at the IM stage, which significantly decreased at the initial maturity stage (MA). In contrast, cultivars S15 and S19 showed relatively low sucrose contents across all stages, indicating distinct sugar metabolic patterns (Figure 2b). The glucose content gradually increased from IM to PM in all germplasms, and cultivar S15 exhibited a significantly higher glucose content at the PM stage than the other cultivars (Figure 2c). Based on the largest difference in sugar contents, cultivars S4 and S19 were selected for subsequent experiments.

### 3.2. RNA-seq Analysis of Fruit Flesh Tissues from Two A. trifoliata Genotypes

To elucidate the molecular mechanisms underlying the sugar metabolism in *A. trifoliata* fruit flesh, pulp samples collected at three developmental stages (IM, MA, and PM) of S4 and S19 germplasms were used for RNA-seq sequencing. The total RNA was extracted and three biological replicates were prepared for each sample, resulting in 18 sequencing libraries (Table 3). In total, 130.87 GB of raw bases were generated, with 46.15–51.25 million raw reads per library. The Phred quality score is a key indicator for evaluating the RNA-seq data quality, and Q30 values above 80% are generally considered reliable. In this study, Q30 values for the 18 libraries ranged from 93.39% to 93.88%, and GC contents ranged from 43.94% to 44.54%, indicating that the sequencing data were of sufficient quality for downstream analyses.

**Figure 3:**
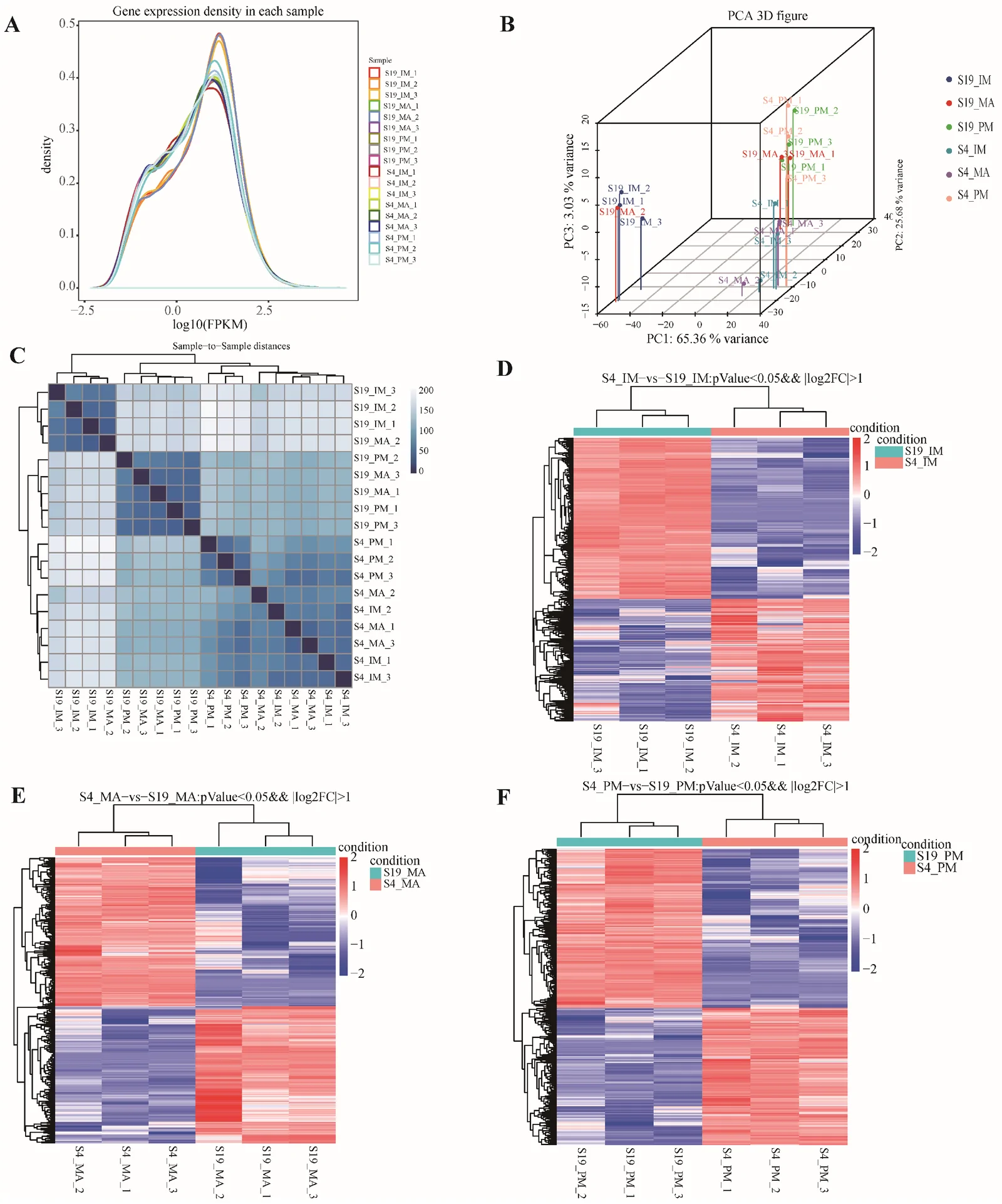
Analysis of transcriptome sequencing results: (**A**) distribution of gene expression abundance across samples; (**B**) principal component analysis (PCA); (**C**) sample correlation heatmap; and (**D**–**F**) clustering of pulp samples in three developmental stages with three replicates. Red indicates up-regulated genes; blue indicates down-regulated genes. Dendrograms on the top and left represent sample-to-sample and gene-to-gene similarities, respectively.

To further assess the cDNA library quality, we examined the unigene expression abundance and distribution patterns across the 18 samples (Figure 3A). The expression abundance curves of all samples showed highly consistent distribution patterns and exhibited a clear unimodal peak. Principal component analysis (PCA) showed that there was a significant separation between the three developmental stages of S4 and S19, and the biological replicates in each group were closely clustered, showing a good repeatability and significant differences between the treatment groups (Figure 3B). Consistently, sample-to-sample hierarchical clustering showed that biological replicates from each developmental stage clustered into the same branch for both the S4 and S9 genotypes, further supporting the high data reproducibility and marked differences among groups (Figure 3C). In order to compare the gene expression differences between S4 (high accumulation type) and S19 (low accumulation type) during fruit development, we performed a hierarchical clustering analysis of differentially expressed genes (DEGs) at three key developmental stages (IM, MA, and PM). The biological replicate samples of the same genotype were clustered in the same branch, indicating that the transcriptome data had good repeatability (Figure 3D–F). These results demonstrate a pronounced divergence in gene expression patterns among the three developmental stages in *A. trifoliata* fruit flesh and a high consistency among biological replicates.

### 3.3. Differentially Expressed Gene Analysis

**Figure 4:**
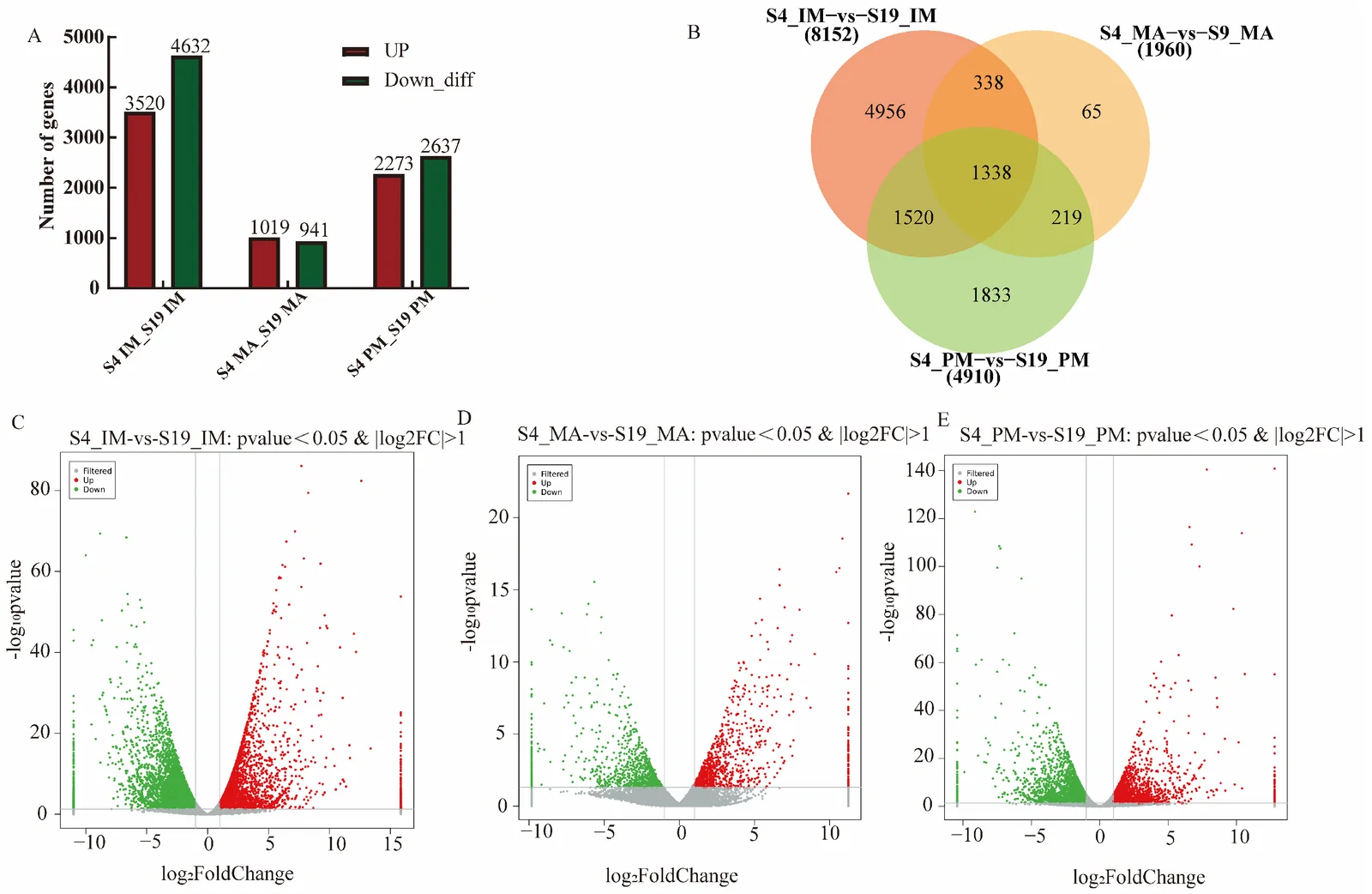
Differentially expressed gene (DEG) analysis: (**A**) bar chart showing the numbers of DEGs in the three comparisons; (**B**) Venn diagram of DEGs among the three comparisons; and (**C**–**E**) volcano plots of DEGs in the three comparisons, showing the distribution of significantly up-regulated, down-regulated, and non-significant genes.

In order to study the differences in transcription levels of the high-fructose genotype S4 and low-fructose genotype S19 at different developmental stages, a differential expression analysis was performed on S4 and S19. Genes with *p*-value < 0.05 and log2foldchange > 1 were defined as differentially expressed genes (DEGs). A total of 10,269 DEGs were identified between S4 and S19; during the IM, MA, and PM stages, the number of DEGs between S4 and S19 was 8152, 1960 and 4910, respectively (Figure 4A).

The Venn diagram analysis showed that 4956 DEGs were unique to the IM comparison group (S4 vs. S19), which was higher than that of the PM and MA comparison groups (1833 and 65). In addition, there were 1338 DEGs in the comparison of IM, PM, and MA (Figure 4B). The volcano map helps to quickly identify genes that are significantly up-regulated or down-regulated under different conditions; in each comparison group, the points closer to the top of the *y*-axis represent a higher statistical significance, while the points closer to both ends of the *x*-axis indicate a larger fold change in expression (Figure 4C–E).

### 3.4. Functional Annotation of DEGs

**Figure 5:**
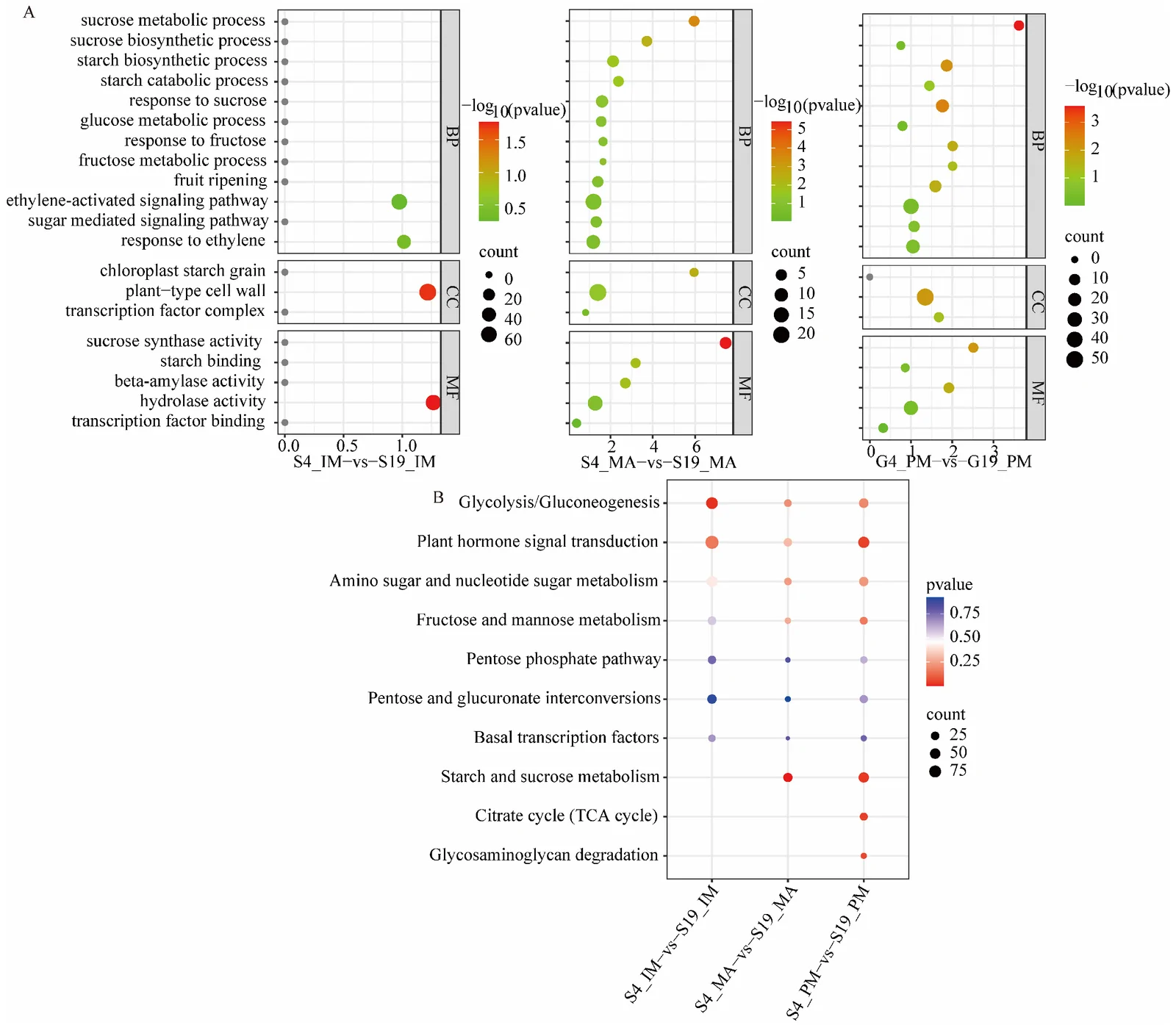
Enrichment analysis of DEGs. GO. (**A**) GO classification and distribution. (**B**) Enriching the most significant 20 KEGG pathways of S4_IM vs. S19_IM, S4_MA vs. S19_MA, and S4_PM vs. S19_PM.

In this study, GO annotation showed that genes from the three comparisons could be assigned to 64 functional categories within the three main Gene Ontology (GO) domains: biological process (BP), cellular component (CC), and molecular function (MF). The GO enrichment analysis showed that most of the annotations were the biological process kind. Most DEGs in the IM comparison group were mainly involved in the response to ethylene, and ethylene-activated signaling pathways, and DEGs in the MA and PM groups were mainly involved in sucrose metabolism, glucose metabolism, and fructose metabolism. The annotation of the plant-type cell is most prominent at the level of the cell component. Notably, the chloroplast starch grain was enriched in the MA comparison group, and the transcription factor complex were enriched in MA and PM. Sucrose synthase activity, starch binding, beta-amylase activity, hydrolase activity, and transcription factor binding are the main categories of molecular functions in MA and PM (Figure 5A), suggesting the stage-dependent regulatory mechanisms of sugar metabolism during *A. trifoliata* fruit development.

The pathway-based analysis provides a deeper understanding of gene function and interaction. Using the KEGG database, 2496 unigenes were mapped to 129 biological pathways. The KEGG enrichment analysis of these DEGs can provide an in-depth understanding of the molecular mechanisms of key metabolic processes during the development of *A. trifoliata*. The top 20 enriched pathways included those directly involved in sugar biosynthesis, such as starch and sucrose metabolism, fructose and mannose metabolism, galactose metabolism, amino sugar and nucleotide sugar metabolism, and the pentose phosphate pathway. In addition, several other pathways associated with sugar metabolism were also enriched, including plant hormone signal transduction, basal transcription factors, pentose and glucuronate interconversions, and the citrate cycle (Figure 5B). These KEGG enrichment results indicate that sugar biosynthetic pathways in *A. trifoliata* fruit flesh undergo substantial regulation during fruit ripening.

### 3.5. WGCNA Co-Expression Network Analysis and Identification of Key Regulatory Genes

**Figure 6:**
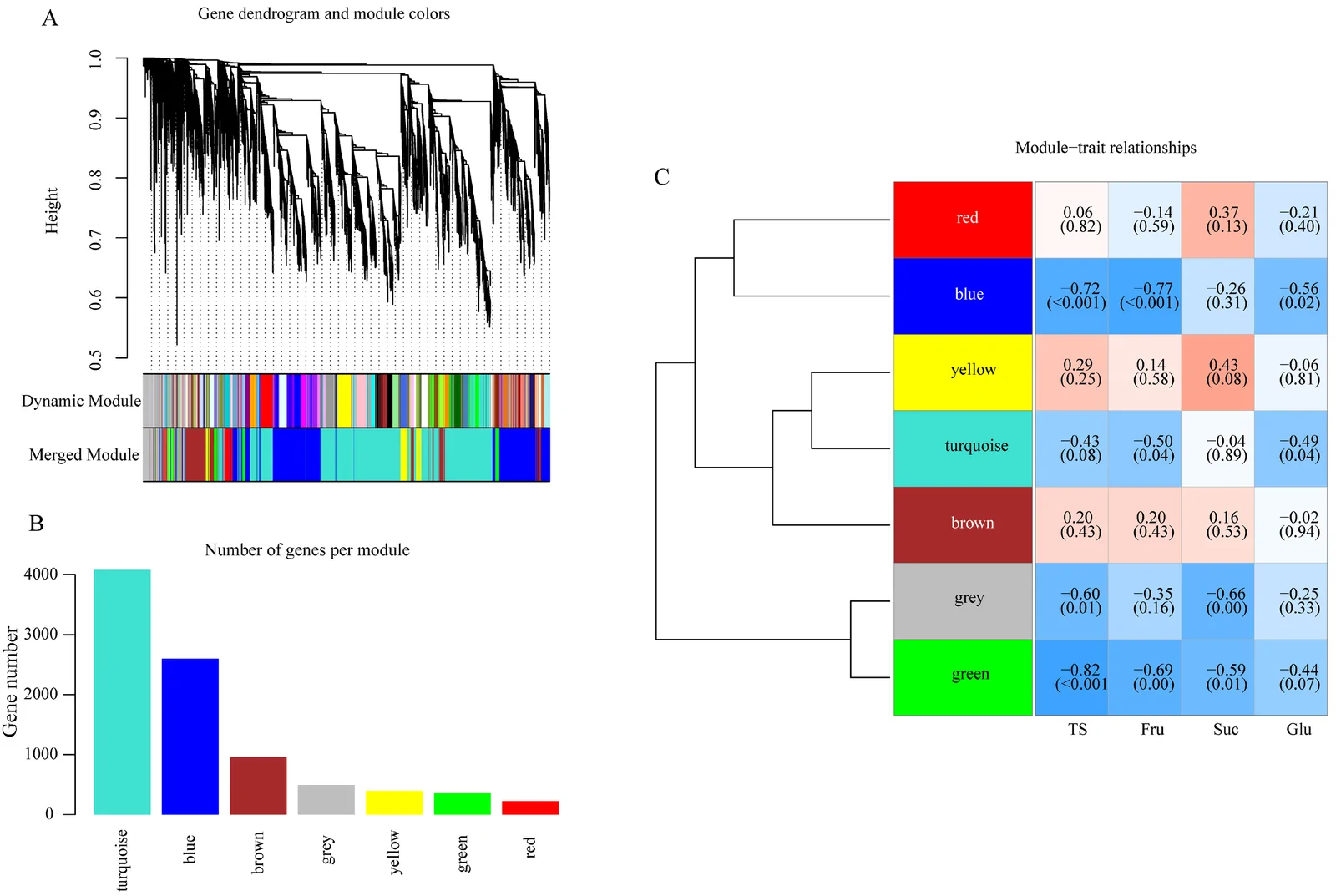
A WGCNA of differential gene expression during *A. trifoliata* development was performed. (**A**) Hierarchical clustering tree diagram of module feature genes and heat map of neighboring relationships in the feature gene network, with each branch representing a module and each leaf representing a gene. Each colored row represents a color-coded module containing a set of highly interconnected genes. (**B**) The number of genes associated with different modules. (**C**) Module eigen–gene relationships with physiological traits and sample relationships are shown. Each row corresponds to a module. TS: total sugar, Fru: Fructose, Glu: glucose, Suc: sucrose. Each column corresponds to a trait. The color of each cell at the row-column intersection indicates the correlation coefficient between the module and the trait, and the numbers in each cell indicate correlation coefficient R and *p* value, respectively.

To identify candidate genes highly related to sugar metabolism during the development of *A. trifoliata* pulp, a weighted gene co-expression network analysis (WGCNA) was performed using transcript abundance combined with changes in glucose, fructose, and sucrose. A total of 9104 genes were identified. Among them, 8633 genes were assigned to six co-expression modules (each marker had a distinct color), and the remaining 471 genes were designated as gray (unassigned). The hierarchical clustering tree and the module-trait association heat map are given in Figure 6. Among these, the turquoise module contained 4079 genes, which was the largest cluster of genes; the smallest module, the red module, only contained 224 genes. Notably, the green and blue modules exhibited a strong correlation with the accumulation patterns of fructose (Fru) and total sugar (TS), with the correlation coefficients being greater than 0.6 (*p* ≤ 0.01). The highest correlation was observed with total sugar (r = −0.82, *p* ≤ 0.001), followed by fructose (r = −0.77, *p* ≤ 0.01). A statistical enrichment analysis of genes in turquoise, green, brown and blue modules was performed to explore the biological functions of transcriptomes in this module (Figure 6C, Table S3). The distribution of these unique genes in the key pathways of glucose metabolism was clarified, including glycoside synthesis/gluconeogenesis, starch and sucrose metabolism, and fructose and mannose metabolism. We speculated that turquoise, green, brown, and blue module genes may be related to sugar metabolism.

### 3.6. qRT-PCR Validation of Differential Gene Expression

**Figure 7:**
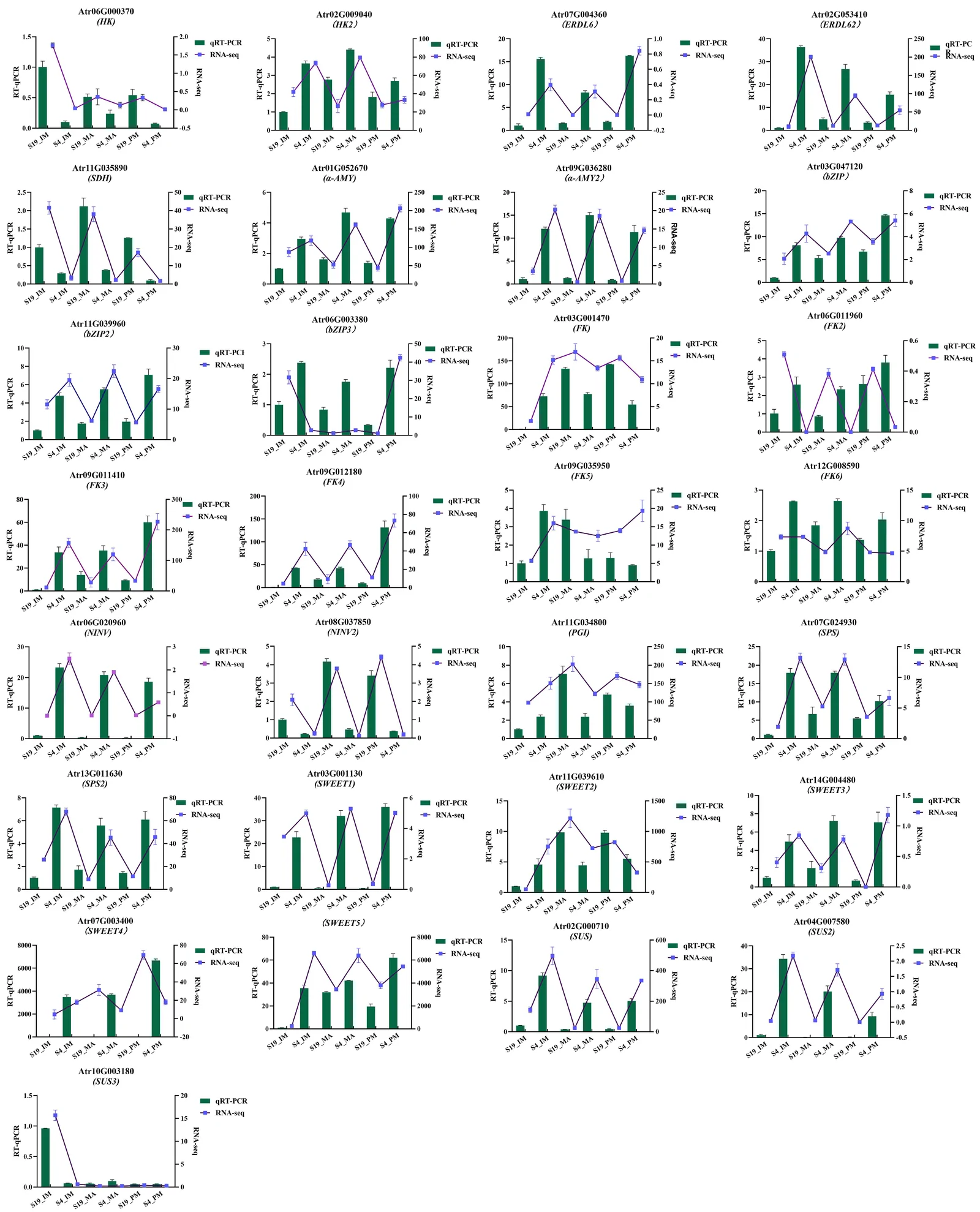
The qRT-PCR was used to validate the DEGs at different developing stages of *A. trifoliata*. The qRT-PCR was used to validate the DEGs at different developing stages of C *A. trifoliata* pulp. The green histogram in the figure represents the qRT-PCR results, and the lines represent the fold change values in the RNA-seq results of S4 and S19 in IM, MA, and PM stages.

To verify the reliability of the RNA-seq data and construct a regulatory network related to soluble sugar metabolism, 29 DEGs that may be involved in sugar metabolism were selected for a quantitative real-time PCR (qRT-PCR) to detect the gene expression in these modules (Figure 7). The qRT-PCR analysis showed that the expression levels of genes such as fructokinase (*FK*), neutral invertase (*NINV*), sucrose synthase (*SUS*), sugar transporter ERD6-like 7 (*ERDL6*), SWEET transporter (*SWEET*), sucrose phosphate synthase (*SPS*), alpha-amylase (*AMY*), and bZIP transcription factor (*bZIP*) in S4 (high sugar accumulation) were significantly higher than those in S19 (low sugar accumulation) germplasm, and the expression levels were low at the IM stage and peaked at the PM stage, indicating that the expression levels of these genes increased with fruit ripening, which was consistent with the RNA-seq findings, thereby confirming the reliability of the transcriptome data. These results indicate that these genes are involved in sugar synthesis and transport, and are highly correlated with sugar accumulation.

### 3.7. Differential Expression Patterns of Genes Involved in Key Sugar Metabolic Pathways

**Figure 8:**
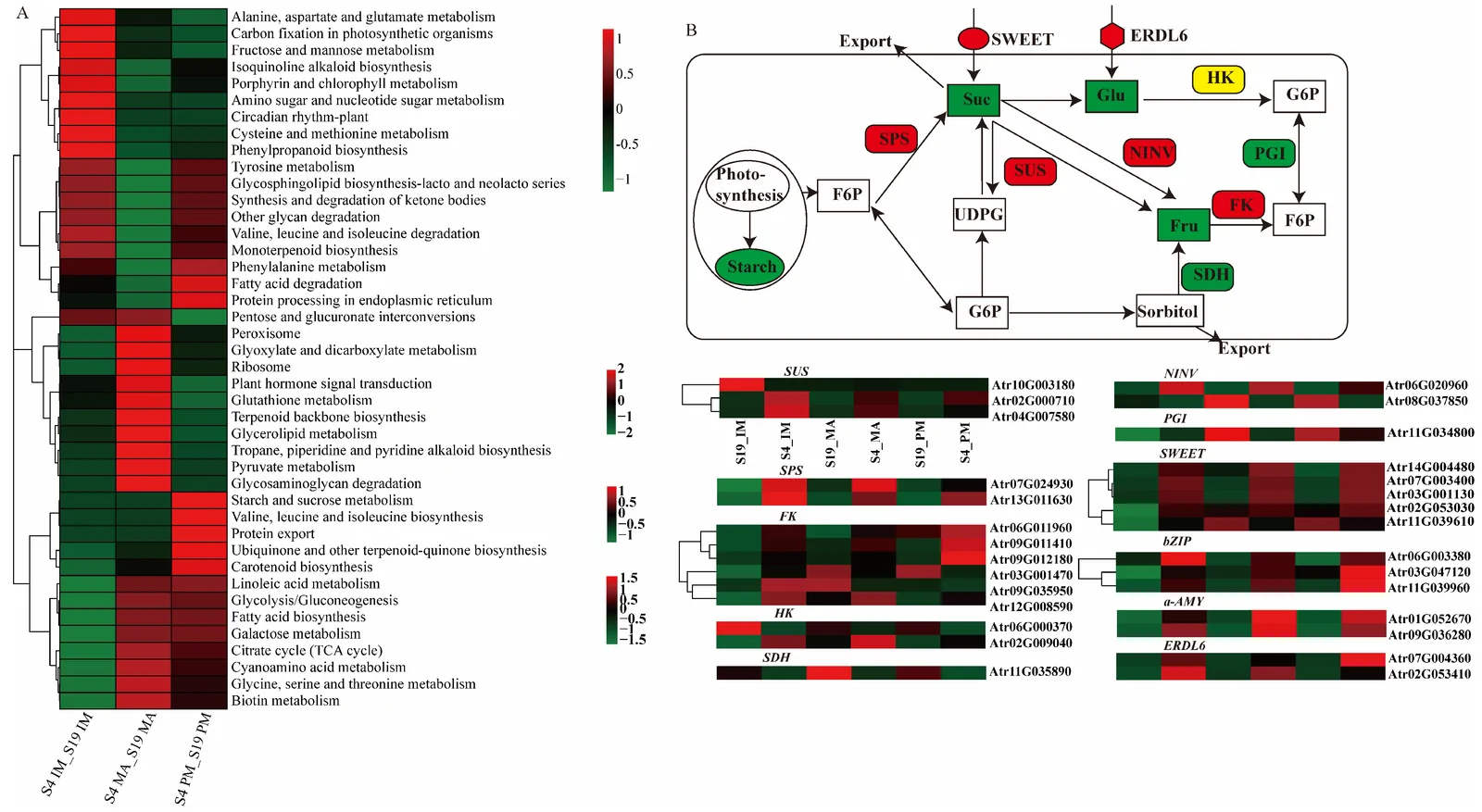
Key gene expression patterns of sugar metabolism pathways in *A. trifoliata* pulp: (**A**) hierarchical clustering of KEGG pathways showing the differential pathway patterns at IM, MA, and PM stages; and (**B**) expression heatmap of key genes in the starch and sucrose metabolism pathway. SUS: Sucrose synthase; SPS: Sucrose-phosphate synthase; FK: Fructokinase; HK: Hexokinase; SDH: Sorbitol dehydrogenase; NINV: Alkaline/Neutral invertas, PGI: Phosphoglucose isomerase; SWEET: SWEET sugar transporter; bZIP: Basic leucine zipper; α-AMY: Alpha-amylase; ERDL6: Sugar transporter ERD6-like; F6P: Fructose-6-phosphate; G6P: Glucose-6-phosphate; UDPG: Uridine diphosphate glucose; Suc: Sucrose; Glu: Glucose; Fru: Fructose.

In this study, based on the FPKM values and qRT-PCR analysis, we performed a hierarchical clustering of genes in the three comparison groups and an expression profile analysis of genes related to key pathways of sugar metabolism, of which 29 genes were differentially expressed during fruit development (Figure 8). In the three developmental stages of *A. trifoliata* pulp, the expression of single genes encoding key enzymes of sucrose and starch metabolism reached the peak. In the three developmental stages, the expression of *SS* and *SPS* reached the peak in the IM stage. Furthermore, the expression levels of *AMY* and *bZIP* genes demonstrated the maximal expression in the MA stage of S4 germplasm. Genes encoding key enzymes in fructose and sugar transport, such as *FK*, *NINV,* and *SWEET,* showed a significant increase in expression during fruit development and peaked at maturity. In contrast, the expression of the *ERDL62* and sorbitol-dehydrogenase (*SDH*) was significantly down-regulated during the PM stage. These results highlight the significant differences in the regulatory mechanisms of sugar-metabolism-related genes at different developmental stages, indicating that they have different biological roles in coordinating the energy metabolism and biosynthetic demands at different growth stages.

## 4. Discussion

Sugar accumulation is a complex biological process, which mainly involves the synthesis and transport of sugars. However, the mechanism of sugar accumulation in *A. trifoliata* is still unclear. RNA-seq is a powerful approach for dissecting transcriptional events underlying sugar biosynthesis. The mechanism of sugar-acid accumulation during pomegranate fruit development was studied by RNA-seq, and a gene network related to sugar-acid ratio accumulation was constructed, which proved that transcriptional regulation was closely related to sugar metabolism [32]. Through transcriptome analysis, DEGs related to soluble sugar and organic acid accumulation were identified in watermelon fruit, which provided a molecular basis for understanding the quality of sweet fruit [6]. In this work, the RNA-seq analysis of *A. trifoliata* fruit flesh from different genotypes and developmental stages enabled a more comprehensive characterization of the expression dynamics and facilitated the monitoring of transcriptional regulation across ripening. The constructed 18 sequencing libraries were aligned to the reference genome of *A. trifoliata*, and 10,269 DEGs were identified. The differentially expressed genes between S4 and S19 were the most in the IM stage, followed by the PM stage, indicating that the early and mature stages were the key periods for sugar-metabolism-related transcriptional reprogramming. The GO functional analysis further showed that DEGs were mainly enriched in biological process terms (cellular processes) and molecular function terms (e.g., catalytic activity and binding). The KEGG functional enrichment analysis showed that these DEGs were involved in pathways such as starch and sucrose metabolism, fructose and mannose metabolism, and galactose metabolism, which was consistent with the observed changes in the pulp sugar content during ripening.

Genes often participate in biological processes through co-expression. WGCNA is a powerful tool for constructing gene co-expression networks and calculating module–trait relationships. Therefore, this study used WGCNA to construct a co-expression network to identify key unigenes involved in sugar metabolism during *A. trifoliata* fruit ripening, including *SUS*, *SPS*, *FK*, *NINV*, and *SWEET*. The results of qRT-PCR showed that these genes related to sugar metabolism and transport showed different regulation in different germplasm and different developmental stages. The soluble sugars in the fruits of *A. trifoliata* are mainly composed of glucose, fructose, and sucrose. The content and proportion of soluble sugars are different among different tree species and different developmental stages of the same tree species. The high concentration of sucrose provides an important basis for nutrient accumulation and quality formation during fruit development [33]. *SUS* can catalyze the reverse conversion of sucrose and UDP to UDP-glucose and fructose [34]. As a key enzyme in sucrose synthesis, *SPS* not only is involved in the synthesis of sucrose, but also has the function of decomposing sucrose into fructose and uridine diphosphate glucose [35]. The results showed that the expression levels of *SUS* and *SPS* genes were higher in the IM stage, and the sucrose content was higher during this period, and continued to maintain a high level in the MA and PM stages. This may be due to the fact that *SUS* and *SPS* genes mainly act on the decomposition of sucrose and increase the activity of its related enzymes, thus accelerating the decomposition of sucrose, which likely promotes sucrose hydrolysis into fructose and glucose and thereby directly enhances fruit sweetness [8]. Similar mechanisms have been reported during ripening in fruits such as strawberry, peach, and watermelon, where sucrose degradation and sugar transport pathways play critical roles in sweetness formation [18,19,20].

Neutral/alkaline invertase (*NINV*) irreversibly catalyzes the conversion of sucrose into glucose and fructose, and plays an important regulatory role in plant growth and development [36]. The sugars will finally be exported; transporters (*SWEETs*) play a crucial role in sugar transport pathways, which can promote the bidirectional transport of sugar, including glucose, fructose, and sucrose [37]. In this study, *NINV*, associated with sucrose cleavage into glucose and fructose, was markedly enhanced in S4 at the PM stage; *SWEET*, associated with transmembrane sugar transport, was significantly up-regulated in S4 at the PM stage; whereas *ERDL6*, associated with vacuolar sugar transport, was higher in S4 at IM and MA. Together, these patterns suggest that S4 more actively coordinates sugar “synthesis–conversion–transport/mobilization” during development. This is consistent with previous findings in fruit sugar research showing that SPS/SUS/invertases and sugar transporters participate in sugar accumulation and partitioning [38,39].

Meanwhile, the starch branch also exhibited clear stage-dependent differences: starch synthase (SS) showed a stage-specific enhancement at MA and PM, whereas starch-degradation-related BAM/AMY displayed strong up-regulation in S4 at MA, implying more active starch mobilization during this period and providing substrates for the subsequent soluble sugar accumulation [40]. Fructokinase (FK) is a regulator of fructose signaling in plants and gateway proteins that catalyze the initial step in fructose metabolism through phosphorylation [41]. Moreover, FK family genes involved in fructose utilization showed gene-specific inconsistency but an overall stronger expression tendency in S4 at PM, suggesting that S4 may more actively channel fructose into downstream metabolism during late development; this is consistent with the key role of fructose phosphorylation in controlling carbon flux during fruit development [42]. The bZIP transcription factors (TFs) are known to regulate fruit sugar accumulation [43]. Moreover, the sugar-signaling-related *bZIP* was significantly enhanced in S4 at MA, implying a mid-stage regulatory response aligned with sugar level changes, which may partially explain the coordinated expression of sugar metabolism genes at later stages. These results indicated that the effects and mechanisms of different sugar-metabolism-related genes on the soluble sugar content and glucose, fructose, and sucrose accumulation were different. Especially in the late stage of sugar accumulation, the up-regulation of sugar transporters and sugar metabolic enzymes may play a key role in regulating the sugar accumulation and final fruit quality. Therefore, a further analysis can be performed on the gene action mode and deep molecular mechanism related to the difference of sugar accumulation among different varieties of *A. trifoliata*, and provide a reference for analyzing the influence mechanism of berry fruit traits.

## 5. Conclusions

A transcriptome analysis was performed on the pulp of *A. trifoliata* across different developmental stages and germplasms, and 29 differentially expressed genes (DEGs) associated with sugar metabolism were validated by qRT-PCR. The results revealed that the expression levels of sugar-metabolism-related genes—including the *SUS*, *SPS*, and *SWEET* family—were significantly higher in the high-sugar accumulation germplasm (S4) than in the low-sugar accumulation germplasm (S19). Furthermore, an expression pattern analysis demonstrated substantial variation in the sugar metabolism pathway genes between varieties and across developmental stages. These findings provide a critical physiological and molecular foundation for understanding the regulatory mechanisms governing sugar metabolism in *A. trifoliata* fruits. Moreover, the identification of key sugar metabolism genes and their associated regulatory networks offer potential targets for future molecular breeding and genome editing strategies aimed at enhancing the sweetness and nutritional quality of *A. trifoliata* fruit, thereby supporting its development as a functional food ingredient with an improved market value.

## Figures and Tables

**Table 1:** Primers and their sequences used for qRT-PCR.

ID	Primer	Primer Sequence (5′ to 3′)
1	Atr03G001470-F:	AGTTGGTGGGACTGCTC
2	Atr03G001470-R:	GGACTGATGTATCGGTTCT
3	Atr06G011960-F:	TTGCAGGGTATAGTGGC
4	Atr06G011960-R:	GCTGATGGCATAGTTCC
5	Atr09G011410-F:	CTGCTAAGGCTGAAGGTA
6	Atr09G011410-R:	ATCGTCTGCGGAATCTC
7	Atr09G012180-F:	GTTGTTATGTCCCTGTGGT
8	Atr09G012180-R:	TCCTTTCTGGGTTGTGC
9	Atr09G035950-F:	TTGAACCTGGGAAAGTC
10	Atr09G035950-R:	AGCACTACCACGAGAAAC
11	Atr12G008590-F:	CGTCTCACGCCAACCAC
12	Atr12G008590-R:	TCGAGAACGAAACCTAAT
13	Atr11G035890-F:	GCATCGGAATAAGCAAC
14	Atr11G035890-R:	CAGGGTCATCCAAACAT
15	Atr06G020960-F:	TTCGCATTAGGTAACTGT
16	Atr06G020960-R:	CATCTTGTATTCTTAGGGTC
17	Atr08G037850-F:	AAGCAAATCCCAAGAGC
18	Atr08G037850-R:	ATCCGAGTATAATTCCAAAC
19	Atr02G000710-F:	TGTTCTGGGCTATCCTG
20	Atr02G000710-R:	ACTCCCTTCTCATTTCTG
21	Atr04G007580-F:	GCCAGTTTACAGCGGATTT
22	Atr04G007580-R:	GGTGGAGGGTTGTGAGT
23	Atr07G024930-F:	TAGAGTATTGGGTGAACAGAT
24	Atr07G024930-R:	TCCGCCTCCTTATCTTG
25	Atr10G003180-F:	TCTAATGGCGGAACCTA
26	Atr10G003180-R:	CTCCTCCTTGAGTAATCTG
27	Atr11G034800-F:	GGACGCCAACTGCGATTT
28	Atr11G034800-R:	AACATTCCATACGCTCAA
29	Atr13G011630-F:	TAAGGCTGAGGAGGTGA
30	Atr13G011630-R:	AGAGTATTATTGGGTCGTT
31	Atr02G053030-F:	CGTATTTGCCGCTCCTC
32	Atr02G053030-R:	CGTCCTTGGTGCCCTTG
33	Atr07G003400-F:	TTGGAAATCTTGCGTCTC
34	Atr07G003400-R:	TCACCTTCCCTTTGTCG
35	Atr01G052670-F:	TGCCTGGAAGGTTGTAT
36	Atr01G052670-R:	CCCTTTACCGTCAGAAT
37	Atr09G036280-F:	ATACGCCAAGGGACAGG
38	Atr09G036280-R:	TCGCCAAGAACTTTGATGC
39	Atr02G009040-F:	GCTGCTGTTATCTTGGG
40	Atr02G009040-R:	AATTCCTGGTCATATTCTGT
41	Atr07G004360-F:	GTGATTCGCAGAGTAGC
42	Atr07G004360-R:	CTCGGATAACGACAAGC
43	Atr03G047120-F:	AGTGGTCCCTACTGCTCTT
44	Atr03G047120-R:	ACATTCCGCCTGCTTAC
45	Atr11G039960-F:	TTTGGAAGTGAAGAGCAGGTA
46	Atr11G039960-R:	GCAGGGTTGTTTGGAGC
47	Atr14G004480-F:	GCCTTAGTGTTCTTCTGTC
48	Atr14G004480-R:	ATTGTCTCCGCACCATC
49	Atr11G039610-F:	GTATCGGTGGTGGTTGG
50	Atr11G039610-R:	CAGGCTGGCTCACTAAA
51	Atr02G053410-F:	GAGCCATTTATCCAACA
52	Atr02G053410-R:	GACCCGCAAACTACAAC
53	Atr03G001130-F:	AGGACCCTCATACTCGG
54	Atr03G001130-R:	GTCCATTCGCATAAACC
55	Atr06G003380-F:	TCGGTCCTTGGTTAGAG
56	Atr06G003380-R:	GGTAGAAACATTGGCAGTC
57	Atr06G000370-F:	GTGACGGTAGAGGGTGG
58	Atr06G000370-R:	TGAAGCAGCGAGAAACA

**Table 2:** qRT-PCR reaction system and procedure.

Reaction System			Reaction Procedure		
Name	Volume (µL)	Step	Temperature	Time	Cycles
		Initial denaturation	95 °C	1 min	1
SYBR Green qPCR	5	Denaturation	95 °C	20 s	40
Primer F/R	0.5	Annealing	60 °C	30 s	40
cDNA	1	Extension	72 °C	30 s	40
ddH_2_O	Up to 10	Final extension	72 °C	10 min	1

**Table 3:** Sequencing statistics for *A. trifoliata*.

Sample	RawReads (million)	RawBases (Gb)	CleanReads (million)	CleanBases (Gb)	ValidBases (%)	Q30 (%)	GC (%)
S19_IM_1	49.63	7.45	48.44	6.70	89.98	93.67	44.04
S19_IM_2	47.54	7.13	46.34	6.45	90.51	93.60	44.09
S19_IM_3	47.07	7.06	45.89	6.38	90.43	93.65	44.05
S19_MA_1	49.58	7.44	48.47	6.75	90.70	93.77	44.22
S19_MA_2	50.74	7.61	49.47	6.96	91.43	93.63	43.94
S19_MA_3	47.47	7.12	46.39	6.44	90.43	93.78	44.30
S19_PM_1	47.83	7.17	46.57	6.40	89.20	93.62	44.12
S19_PM_2	49.04	7.36	47.92	6.60	89.78	93.88	44.23
S19_PM_3	49.70	7.45	48.65	6.69	89.74	93.77	44.34
S4_IM_1	48.50	7.27	47.40	6.58	90.46	93.71	44.37
S4_IM_2	47.94	7.19	46.94	6.53	90.85	93.79	44.33
S4_IM_3	51.25	7.69	50.15	6.97	90.71	93.76	44.54
S4_MA_1	47.18	7.08	46.19	6.45	91.13	93.78	44.33
S4_MA_2	46.41	6.96	45.43	6.31	90.70	93.39	44.22
S4_MA_3	47.53	7.13	46.54	6.50	91.15	93.47	44.18
S4_PM_1	50.68	7.60	49.46	6.83	89.85	93.59	44.30
S4_PM_2	46.15	6.92	45.16	6.26	90.47	93.79	44.41
S4_PM_3	48.25	7.24	47.20	6.51	90.02	93.79	44.39

Q30 content is the percentage of bases with a quality score greater than 30, indicating a base call accuracy of 99.9%. GC content is the percentage of guanine (G) and cytosine (C) bases among the total number of bases.

## Data Availability

Data will be made available on request.

## Supplementary Materials

The following supporting information can be downloaded at: https://www.mdpi.com/article/10.3390/life16091459/s1, Figure S1. The analysis of mean-connectivity curves and soft threshold: (A) connectivity distribution; (B) scale-free topology fit; (C) scale free topology model fit; and (D) mean connectivity; Table S1. Sample test results; Table S2. Statistical results of reads and reference genome alignment rate; Table S3. Statistical enrichment analysis of DEGs within the turquoise, green, and blue modules; Table S4. Fructose, glucose, and sucrose content determination, and variance analysis.
