# Supplementary material for: Comparative Transcriptomic Analysis Reveals Sugar Metabolism and Accumulation Mechanisms in Akebia trifoliata Fruits
Source: Life (Basel). 2026 Aug 31;16(9):1459. doi: 10.3390/life16091459 (PMC13608637; doi:10.3390/life16091459)
Supplement: Supplementary file 1 [file life-16-01459-s001.zip › Figure S1.pdf]

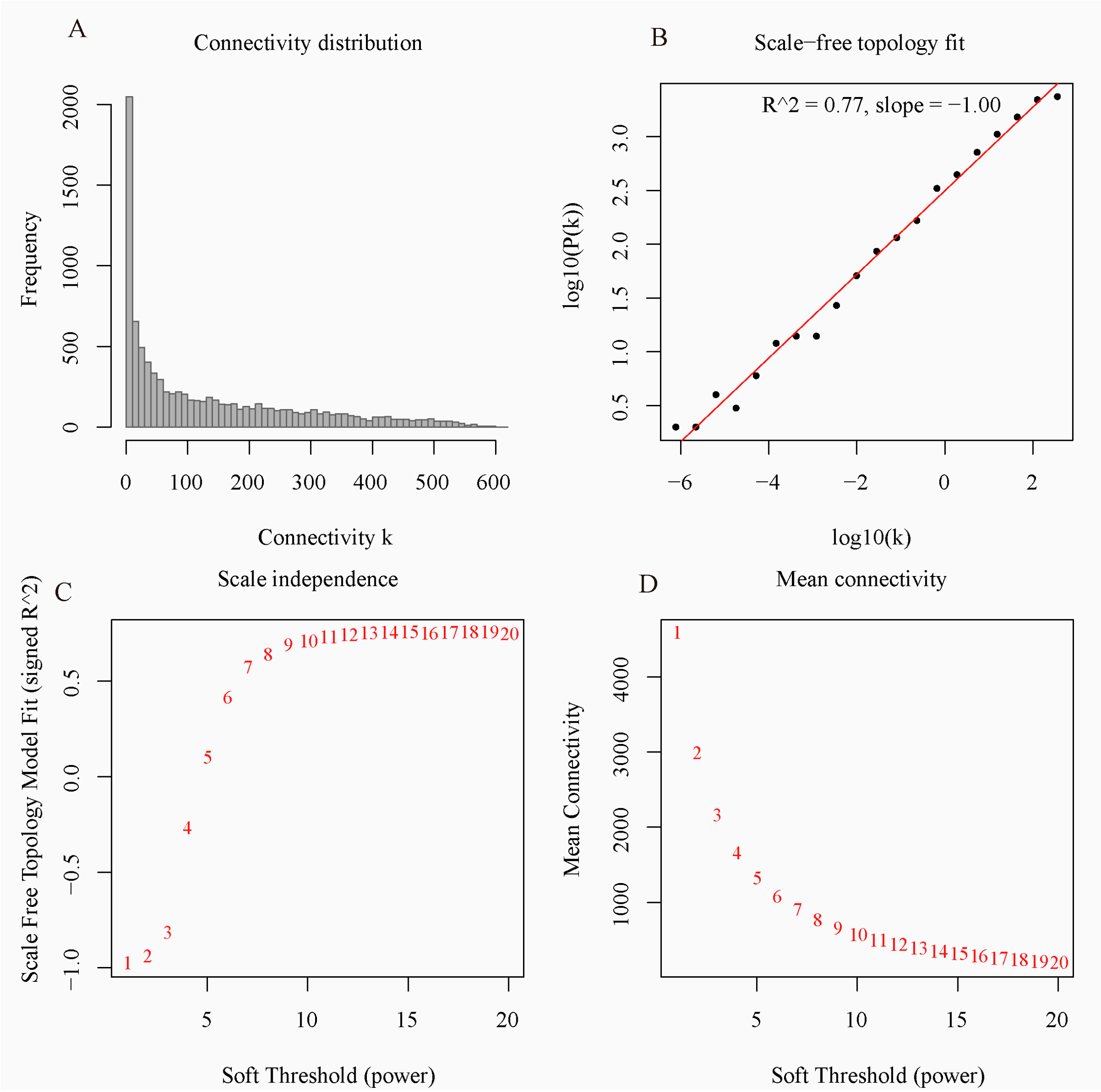

Figure S1. The analysis of mean-connectivity curves and soft threshold: (**A**) connectivity distribution; (**B**) scale-free topology fit; (**C**) scale free topology model fit; and (**D**) mean connectivity.
